# Bioinformatic identification of hub genes *Myd88* and *Ccl3* and TWS-119 as a potential agent for the treatment of massive cerebral infarction

**DOI:** 10.3389/fnins.2023.1171112

**Published:** 2023-05-10

**Authors:** Ai Guo, Bin Gao, Mengting Zhang, Xiaoyu Shi, Weina Jin, Decai Tian

**Affiliations:** ^1^Department of Neurology, Beijing Tiantan Hospital, Capital Medical University, Beijing, China; ^2^China National Clinical Research Center for Neurological Diseases, Beijing Tiantan Hospital, Beijing, China

**Keywords:** massive cerebral infarction, microarray, connectivity map, hub gene, inflammation

## Abstract

**Background:**

Massive cerebral infarction (MCI) causes severe neurological deficits, coma and can even result in death. Here, we identified hub genes and pathways after MCI by analyzing microarray data from a murine model of ischemic stroke and identified potential therapeutic agents for the treatment of MCI.

**Methods:**

Microarray expression profiling was performed using the GSE28731 and GSE32529 datasets from the Gene Expression Omnibus (GEO) database. Data from a sham group (*n* = 6 mice) and a middle cerebral artery occlusion (MCAO) group (*n* = 7 mice) were extracted to identify common differentially expressed genes (DEGs). After identifying gene interactions, we generated a protein-protein interaction (PPI) network with Cytoscape software. Then, the MCODE plug-in in Cytoscape was used to determine key sub-modules according to MCODE scores. Enrichment analyses were then conducted on DEGs in the key sub-modules to evaluate their biological functions. Furthermore, hub genes were identified by generating the intersections of several algorithms in the cytohubba plug-in; these genes were then verified in other datasets. Finally, we used Connectivity MAP (CMap) to identify potential agents for MCI therapy.

**Results:**

A total of 215 common DEGs were identified and a PPI network was generated with 154 nodes and 947 edges. The most significant key sub-module had 24 nodes and 221 edges. Gene ontology (GO) analysis showed that the DEGs in this sub-module showed enrichment in inflammatory response, extracellular space and cytokine activity in terms of biological process, cellular component and molecular function, respectively. Kyoto Encyclopedia of Genes and Genomes (KEGG) analysis revealed that TNF signaling was the most enriched pathway. *Myd88* and *Ccl3* were identified as hub genes and TWS-119 was identified as the most potential therapeutic agent by CMap.

**Conclusions:**

Bioinformatic analysis identified two hub genes (*Myd88* and *Ccl3*) for ischemic injury. Further analysis identified TWS-119 as the best potential candidate for MCI therapy and that this target may be associated with TLR/MyD88 signaling.

## 1. Introduction

Massive cerebral infarction (MCI) is a life-threatening subtype of stroke that can result in fatal intracranial hypertension caused by cerebral edema. Most patients die from cerebral herniation within 1 week and the one-year mortality rate associated with this condition can reach 70–80% (Zhang et al., 2018; Huang et al., 2021). Conventional medical treatment mainly includes osmolar therapies and sedation medicine, such as mannitol, diuretics and propofol, although these therapeutics have many limitations with regards to reversing herniation (Halstead and Geocadin, 2019). Notably, decompressive craniectomy is a lifesaving approach that can be used to reduce intracranial pressure (Neugebauer and Jüttler, 2014). However, decompressive craniectomy is associated with various complications, including seizures, cerebrospinal fluid disturbances, external brain herniation and trephined syndrome (Lin and Frontera, 2021). Moreover, excitotoxicity, calcium influx, inflammatory responses, and oxidative stress are all known to be involved in the pathophysiological processes that occur after MCI; these can cause secondary brain injury and exacerbate clinical outcomes (Shi et al., 2019; Stegner et al., 2019; Yuan et al., 2021; Zong et al., 2022). However, there is still no approved therapeutic drug that can target these processes in clinical practice. Hence, there is an urgent need to identify the molecular mechanisms underlying the pathophysiological processes that take place after MCI and identify potential new therapeutic targets.

Over recent years, microarray technology and bioinformatic analyses have been widely applied to identify differentially expressed genes (DEGs) and functional pathways, thus enhancing our understanding of the mechanisms underlying disease occurrence and development at the molecular level (Hoffmann et al., 2016). Some panels of RNA have been detected in patient blood samples and have assisted in defining the etiology of stroke, such as distinguishing cardioembolic stroke from large-vessel stroke, with >90% sensitivity and specificity (Xu et al., 2008; Jickling et al., 2010). Connectivity Map (CMap) is an algorithm that has become one of the most comprehensive and systematic approaches for analyzing drug-disease associations (Lamb et al., 2006; Subramanian et al., 2017). CMap encompasses gene expression profiles in response to 1,309 agents used to treat human cell lines (Zhang et al., 2017). Consequently, CMap reduces the cost of drug screening while increasing efficiency (Liang et al., 2022). For example, in a previous study, CMap was used to identify luteolin as a potential therapeutic for ischemic stroke; this agent caused effect by activating the PI3K/Akt signaling pathway (Luo et al., 2019). However, this strategy has yet to be used to identify potential agents for the treatment of stroke.

The middle cerebral artery occlusion (MCAO) model is a classic mouse model of ischemia that mimics human MCI and develops a serious edema at 24 h after reperfusion (Pillai et al., 2009). In this study, we aimed to identify hub genes and biological functions related to the pathophysiological processes that occur in the brain after ischemic injury to help elucidate the molecular mechanisms involved in MCI. Using this information, we also aimed to identify potential therapeutic agents for the future treatment of MCI.

## 2. Methods

### 2.1. Data collection

Two microarray expression profiling datasets (GSE28731 and GSE32529) were downloaded from the GEO database (https://www.ncbi.nlm.nih.gov/geo/). The RNA expression profiles of a MCAO model group without therapy along with a sham group were then obtained from the GSE28731 dataset (three MCAO mice and two sham mice) and GSE32529 (four pairs of MCAO and sham mice) (Vartanian et al., 2011; Barreto et al., 2012). Both datasets were based on the GPL1261 Affymetrix Mouse Genome 430 2.0 Array.

### 2.2. Identification of DEGs

Differentially expressed RNAs between the MCAO and sham groups were identified as DEGs by the limma (Linear Models for Microarray Analysis) package in R (GEO2R; http://www.ncbi.nlm.nih.gov/geo/geo2r). The limma package was used to apply multiple-testing corrections on *P*-values to help correct for the occurrence of false positives. The Benjamini and Hochberg (false discovery rate) method was then used to adjust P values to limit false positive errors. Volcano plots were then generated using the ggpubr and ggthemes packages in R. An online visualization tool (Draw Venn Diagram; http://bioinformatics.psb.ugent.be/webtools/Venn/) was then used to identify common DEGs and generate a Venn diagram.

### 2.3. PPI network construction

Next, we constructed a PPI network using the Search Tool for the Retrieval of Interacting Genes (STRING) database (http://string-db.org/) with a minimum interaction score (median confidence) of 0.4; this network was then visualized by Cytoscape software (version 3.7.2) (Szklarczyk et al., 2017, 2019). Next, the Molecular Complex Detection (MCODE) plug-in in Cytoscape was used to identify key sub-modules in the PPI network based on node number and MCODE score (Yan et al., 2019).

### 2.4. Enrichment analysis

In order to clarify potential molecular mechanisms underlying MCI, we used the Database for annotation, visualization, and integration discovery platform (DAVID: http://david.ncifcrf.gov) to perform Gene Ontology (GO) analysis and Kyoto Encyclopedia of Genes and Genomes (KEGG) pathway analysis of the DEGs in the most significant sub-module with a threshold of *P* < 0.05. We then identified the top 10 GO biological processes (BP), the top 10 GO molecular functions (MF), the top10 GO cellular compartments (CC) and the top15 signaling pathways. Bubble maps were then plotted using an online tool (http://www.bioinformatics.com.cn/).

### 2.5. Hub gene identification and validation

Next, the cytohubba plugin in Cytoscape was used to select hub genes by an algorithm-based approach. The intersection of the top 30 genes (calculated by 10 different algorithms) were regarded as hub genes (Deng et al., 2021). Each algorithm was used to score and rank nodes in the PPI network and results were depicted by the UpSetR R package; further validation of the two hub genes was conducted by analyzing the GSE30655 and GSE58720 datasets from the GEO database. A two-tailed unpaired *t*-test following Shapiro-Wilk test was then performed to analyze the difference between the MCAO and sham groups; this was carried out by Graphpad Prism 9.0 and a *P*-value < 0.01 was considered statistically significant.

### 2.6. CMap analysis

Next, mouse gene symbols were converted into human homologs by the homologene package in R. Subsequently, up-regulated and down-regulated DEGs were separately uploaded into the CMap dataset (https://clue.io/). Connectivity scores ranging from −1 to 1 were then calculated by a query signature. A higher negative connectivity score indicated the enhanced ability of a drug to reverse the uploaded expression profiles (Peng et al., 2020). Three agents with the highest negative scores in the compound category were then selected as potential therapeutic agents for MCI.

## 3. Results

### 3.1. Identification of 215 common DEGs

**Figure 1:**
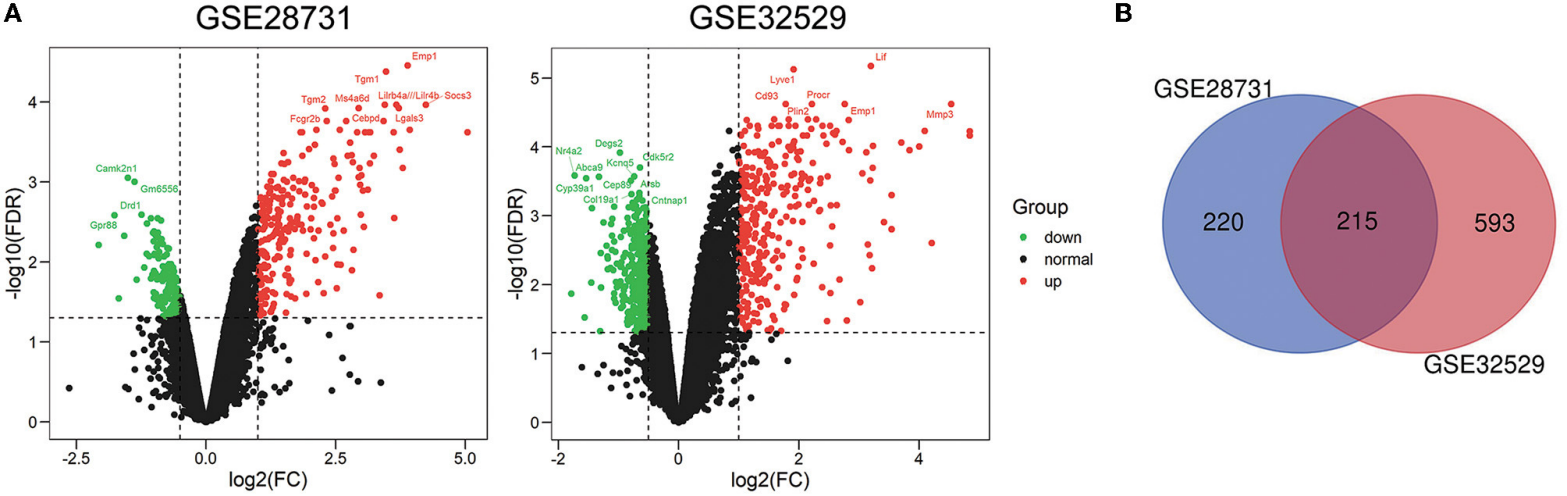
Identification of 215 common differentially expressed genes (DEGs). **(A)** Volcano plots of differentially expressed genes for the GSE28731 and GSE32529 datasets. Red dots represent up-regulated genes and green dots represent down-regulated genes. **(B)** Venn diagram of DEGs between the MCAO and Sham groups; 215 common DEGs were identified.

First, we downloaded the RNA expression profiles of mouse brain tissue at 24 h post-MCAO or following sham surgery from two microarray datasets (GSE28731 and GSE32529) in the GEO database. Further details of the two GSE datasets are given in Supplementary Table 1. We identified 435 DEGs in GSE28731 and 808 DEGs in GSE32529 with a log2(fold-change) ≥1.0 or a log2(fold-change) < -0.5 and an adjusted *P* < 0.05. Subsequently, the DEGs were visualized as a volcano plot (Figure 1A). The intersection of these two datasets featured 215 DEGs, as demonstrated by a Venn diagram (Figure 1B).

### 3.2. Generation of a PPI network and the analysis of sub-modules

**Figure 2:**
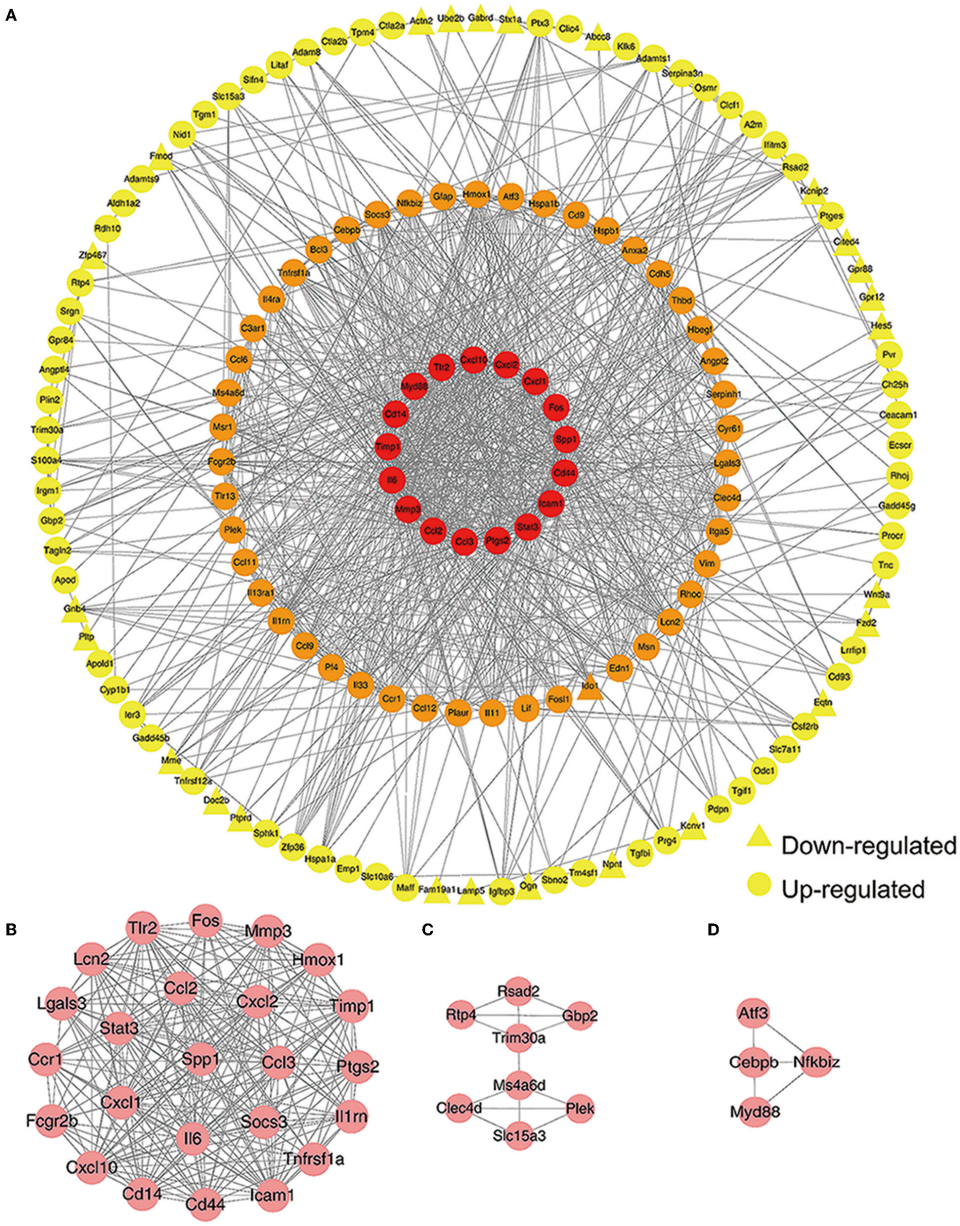
Construction of a protein-protein interaction (PPI) network. **(A)** A PPI network was created by Cytoscape 3.7.2. Red nodes represent a degree >30. Orange nodes represents a degree between 10 and 30. Yellow nodes represent a degree ≤10. Circles represent up-regulated genes while triangles represent down-regulated genes. **(B)** The most significant sub-module. **(C, D)** The other two key sub-modules identified.

After classifying nodes by degree, we generated a PPI network of DEGs that was composed of 154 nodes and 947 edges (Figure 2A). Furthermore, we used the MCODE plugin to identify highly connected sub-modules; this analysis identified three key sub-modules. There were 24 nodes and 221 edges in the most significant sub-module with an MCODE score of 19.217 (Figure 2B). This sub-module featured cytokines and transcription factors. The other two sub-modules contained eight nodes and 13 edges with an MCODE score of 3.714, and 4 nodes and 5 edges with an MCODE score of 3.333, respectively (Figures 2C, D).

### 3.3. Enrichment of inflammatory response and TNF signaling

**Figure 3:**
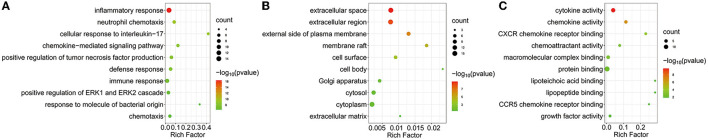
GO function of DEGs in the most significant sub-module. Bubble plot shows the top 10 enriched biological processes **(A)**, cellular components **(B)** and molecular functions **(C)**. The most significantly enriched GO terms are inflammatory response in BP category, extracellular space in CC category and cytokine activity in MF category, respectively. X-axis represents the rich factor (rich factor = the number of DEGs enriched in the pathway/background genes in the pathway). Y-axis refers to pathways. The color and size of each bubble represents statistical significance and the number of DEGs, respectively.

**Figure 4:**
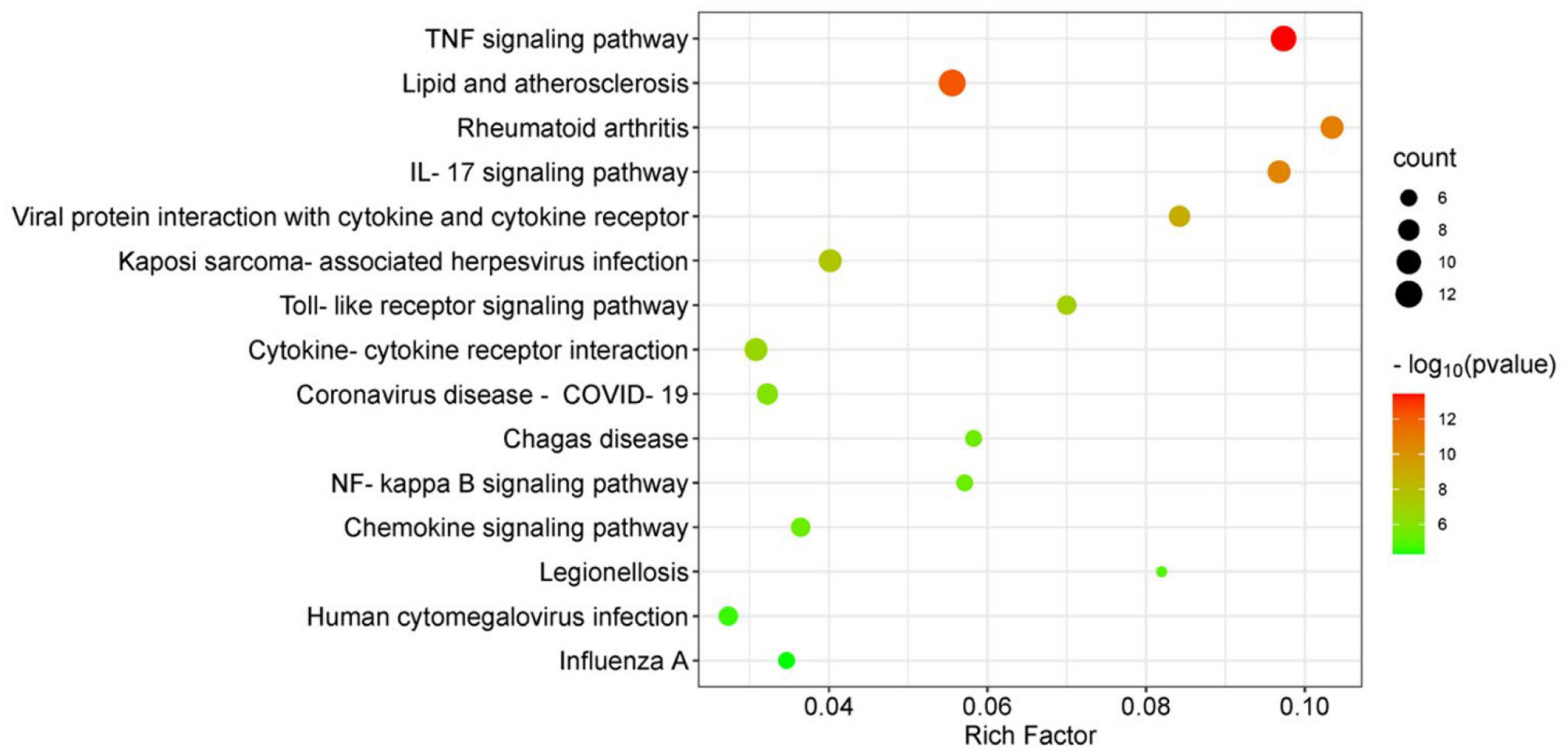
KEGG pathway enrichment analysis of DEGs in the most significant sub-module. Bubble plot shows 15 most-enriched KEGG pathways, among which, TNF signaling pathway is the most significantly enriched KEGG term. X-axis refers to the rich factor, Y-axis refers to pathways. Rich factor indicates degree of enrichment. The bubble color variation from green to red suggests the increased levels of significance of enrichment and the size of the bubbles represents the gene count.

To further study the biological functions of the 24 DEGs in the most significant sub-module, GO term and KEGG pathway analysis was performed. In total, 149 GO items and 46 KEGG pathways were identified (*P* < 0.05). The top 10 GO terms for biological process (BP), cellular component (CC) and molecular function (MF) are depicted in Figure 3. In the BP category, the DEGs were mainly related to cellular response to interleukin-17, the chemokine-mediated signaling pathway, neutrophil chemotaxis, the positive regulation of tumor necrosis factor production, and inflammatory response. In terms of CC, the DEGs were mostly enriched in membrane rafts, the external side of the plasma membrane, the cell surface, the extracellular space, and the extracellular region. With regards to MF, analysis identified enrichment in CXCR chemokine receptor binding, cytokine activity and chemokine activity. The top 15 most enriched KEGG pathways, including the TNF signaling pathway, rheumatoid arthritis, and the IL-17 signaling pathway, are shown in Figure 4.

### 3.4. *Myd88* and *Ccl3* were identified as hub genes and verified by other datasets

**Figure 5:**
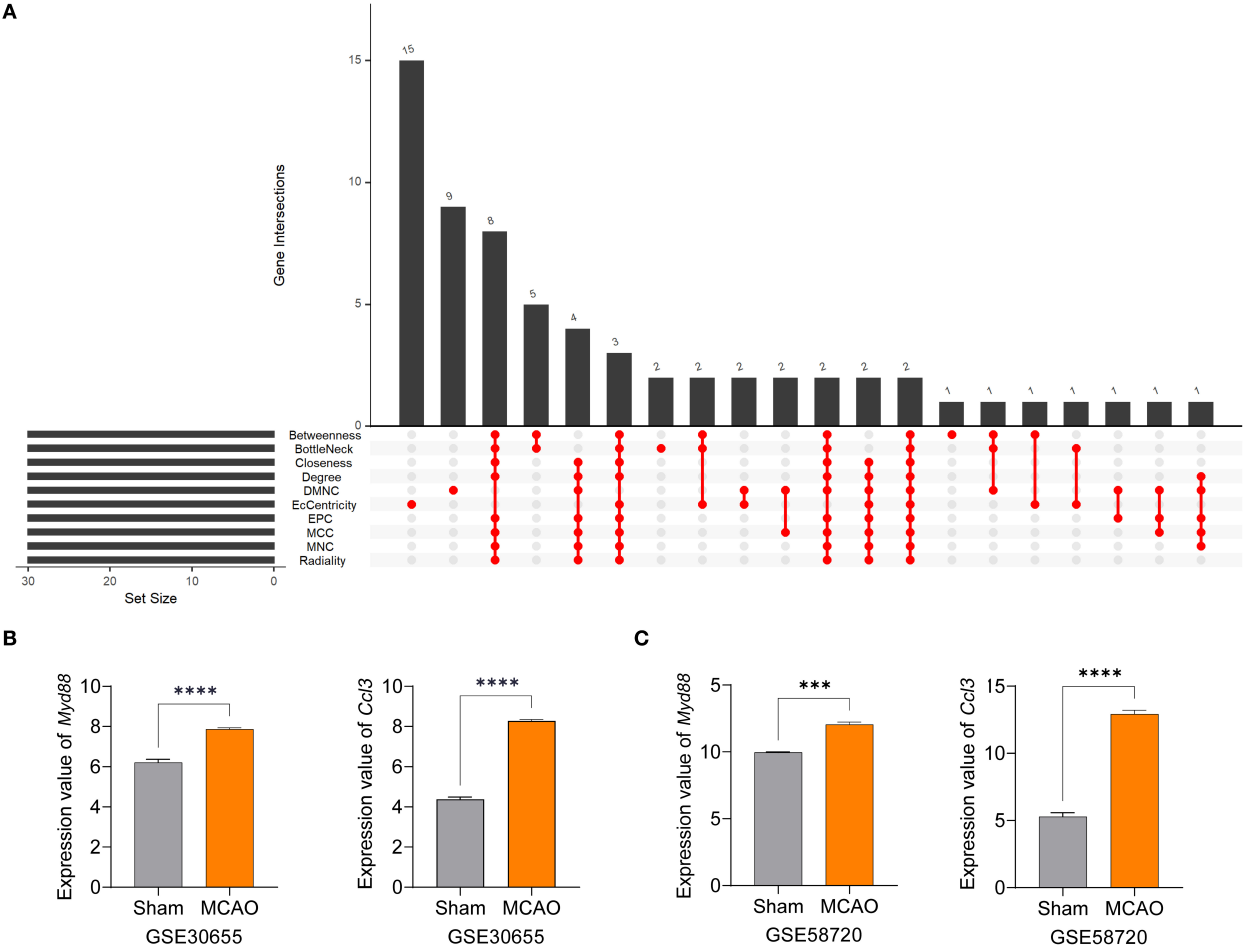
*Myd88* and *Ccl3* were identified as hub genes and verified by other datasets. **(A)** Hub genes were identified by the intersection of 30 genes from 10 algorithms. **(B, C)** The expression levels of *Myd88* and *Ccl3* in the GSE30655 dataset (three sham and seven MCAO mice at 24 h) and the GSE58720 dataset (three sham and three MCAO mice at 50 days). Data fits to normal distribution and are shown as mean ± SEM, ***P < 0.001, ****P < 0.0001.

Myeloid differentiation primary response gene 88 (*Myd88*) and chemokine ligand 3 (*Ccl3*) were identified as hub genes by taking the intersection of 30 genes arising from 10 algorithms in Cytohubba. The ten algorithms were as follows: Betweenness, BottleNeck, Closeness, Degree, Density of Maximum Neighborhood Component (DMNC), EcCentricity, Edge Percolated Component (EPC), Maximal Clique Centrality (MCC), Maximum Neighborhood Component (MNC) and Radiality (Figure 5A). To verify these results, we confirmed the consistent expression levels of hub genes in the GSE30655 dataset which contained three sham samples and seven MCAO samples at 24 h post-surgery (Figure 5B). In addition, we identified high expression levels of the two hub genes in the MCAO group from the GSE58720 dataset which featured three samples in each group at 50 days post-surgery (Figure 5C). These data suggest that *Myd88* and *Ccl3* play key roles in the pathophysiological processes that occur after ischemic injury and that their increased levels of expression may last for several months.

### 3.5. CMap analysis identified TWI-119 as the best potential drug for MCI

To further investigate potential pharmacological therapies for MCI, 116 up-regulated genes and 31 down-regulated genes were uploaded as queries into CMap. Many of these genes are associated with inflammation. Three compounds (TWS-119, lestaurtinib, and tivozanib) had the highest negative enrichment scores and were considered as potential targets for the treatment of MCI. TWS-119 is a form of glycogen synthase kinase (GSK) inhibitor and was the most promising candidate for MCI with a score of −98.77. Lestaurtinib is a janus kinase inhibitor and had a score of −97.92 while tivozanib is a vascular endothelial growth factor receptor inhibitor and had a score of −97.85 (Table 1). These potential agents exerted anti-inflammation effect via their respective pathways.

**Table 1:** The top three compounds in CMap analysis.

**Rank**	**Cmap name**	**Structural formula**	**Mechanisms of action**	**Score**
				
1	TWS-119	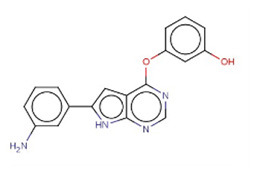	Glycogen synthase kinase inhibitor	−98.77
				
				
2	Lestaurtinib	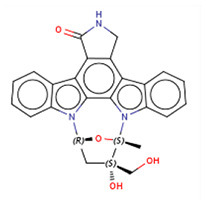	Janus kinase inhibitor, Fms-like tyrosine kinase 3 inhibitor, growth factor receptor inhibitor,	−97.92
				
				
3	Tivozanib	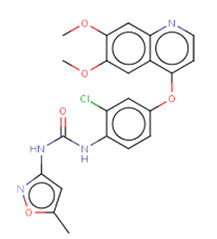	Vascular endothelial growth factor receptor inhibitor	−97.85
				

## 4. Discussion

Massive cerebral infarction (MCI) is a severe cerebrovascular disease associated with high rates of disability and mortality. In the present study, we obtained microarray data relating to MCAO and identified 215 common DEGs. Then, we built a PPI network and determined key sub-modules. Furthermore, GO terms and KEGG pathway enrichment analysis were performed based on the DEGs in most key sub-module and inflammation was identified as an important process occurring after MCAO. Finally, we identified that *Myd88* and *Ccl3* were hub genes and that TWS-119 was the most potential candidate for the therapeutic treatment of MCI.

Inflammatory response was identified as the most significantly enriched BP term along with cytokine activity as the most significant MF term, thus supporting the fact that local inflammatory response after acute stroke arises from the release of proinflammatory cytokines, especially IL-6, IL-1β, TNF and IL-17 (Gulke et al., 2018). Despite satisfactory results in pre-clinical trials, more than 40 clinical trials have been registered in ClinicalTrial and have evaluated anti-inflammatory strategies; however, positive outcomes are limited at the present time (Gulke et al., 2018). KEGG pathway analysis also identified enrichment in TNF signaling. It has been reported that anti-TNF therapy could reduce brain infarct volume and edema in an animal model and improve neurological impairments in patients, thus suggesting that TNF signaling may be a vital inflammatory pathway in acute ischemia (Lambertsen et al., 2019). Thus, there is a clear need to better elucidate the role of neuroinflammation in ischemic stroke.

MyD88 is an adaptor protein that belongs to the toll-like receptor (TLR) and interleukin-1 receptor families. This protein plays a crucial role in the innate immune system, leukocyte activation in inflammatory response, and host defense against Gram-positive bacteria (Kolosowska et al., 2020; Bayer and Alcaide, 2021). TLR4 is known to play a vital role in microglial activation and the inflammatory cascade pathway after CNS injury (Rahimifard et al., 2017). Coincidentally, we identified enrichment in the TLR signaling pathway. In addition, the increased expression levels of *Myd88* in MCAO was confirmed by other datasets. A previous study also reported increased levels of *MYD88* in the peripheral blood of ischemic patients (Li et al., 2020). Another study reported that *Myd88* deficiency in ischemic mice could improve neurological function by attenuating the inflammatory response (Ye et al., 2016). Compared to wild type mice, *Myd88*^−/−^ mice were previously shown to express 7–22% of cytokine and chemokine mRNAs after stab injury (Babcock et al., 2008). However, *Myd88*^−/−^ mice exhibit changes in the functionality of the intestinal barrier and are unable to control bacterial infection (Putnam et al., 2019). Therefore, it is difficult to precisely modulate the expression of *Myd88* in brain ischemia in order to balance its pro-inflammatory and anti-infection properties.

Ccl3, also known as macrophage inflammatory protein-1α (MIP-1α), is considered to be a cardinal element in the regulation of immune cell trafficking and the immune microenvironment in inflammation (Schaller et al., 2017). Ccl3 can also exacerbate brain infarction in the cortical region in MCAO (Takami et al., 2001). Levels of Ccl3 were reported to be elevated in an animal model of stroke and could recruit microglia, neutrophils, and monocytes (Wang et al., 2008; Lee et al., 2016). We also observed the increased expression of *Ccl3* in brain tissue after MCAO. Nevertheless, a previous study failed to find any significant differences in the expression levels of *CCL3* mRNA in peripheral blood mononuclear cells when compared between ischemic stroke patients and a healthy control group (Kostulas et al., 1999), thus suggesting that Ccl3 exerted its chemotaxis function within the CNS after ischemia. CCL3 has been reported to act as a biomarker for fatal events in patients with acute coronary syndrome, thus suggesting a specific role in ischemic injury (de Jager et al., 2012).

TWS119 (4,6-disubstituted pyrrolo-pyrimidine) is a specific GSK-3β inhibitor and has been reported to play a role in reducing brain edema and disruption of the blood-brain-barrier (BBB) in a MCAO model increasing the expression of ZO-1 and claudin-3 (Wang et al., 2016, 2017). Another study showed that TWS119 could ameliorate microglia-mediated neuroinflammation by skewing microglia toward an anti-inflammatory phenotype (Song et al., 2019). Interestingly, it has been reported that the GSK-3β inhibitor can inhibit the TLR4/MyD88 pathway and reduce downstream inflammatory factors in ischemia-reperfusion injury following kidney transplantation (Su et al., 2019). Whether TWS-119 could benefit ischemic injury *via* TLR/MyD88 in a similar manner requires further investigation. Moreover, lestaurtinib, a janus kinase and fms-like tyrosine kinase 3 (FLT3) inhibitor, was shown to attenuate acute lung injury by reducing neutrophil infiltration, which may also attenuate brain edema by inhibiting the JAK2/STAT3 pathway (Li et al., 2019). Tivozanib, a vascular endothelial growth factor receptor (VEGFR) inhibitor, was approved by the United States Food and Drug Administration (FDA) for the treatment of relapsed or refractory advanced renal cell carcinoma (Chang et al., 2022). Vegfr2 mRNA expression was also found to be positively related with brain edema in a diabetic MCAO model, thus supporting our current findings (Kim et al., 2018). Given these points, TWS119 was considered as the most promising therapeutic agent for MCI.

The present study has some limitations that need to be considered. For example, we did not verify the therapeutic efficiency of TWS-119 in MCAO mice. Moreover, further investigation of the precise effects of *Myd88* and *Ccl3* expression in MCI is required.

## 5. Conclusion

In the present study, we identified *Myd88* and *Ccl3* as hub genes and TWS119 as the most potential drug for MCI. Following MCAO, we identified enrichment of the TNF signaling pathway and intense inflammatory responses. Our findings provide new concepts for future research on the precise molecular mechanisms underlying MCI.

## Data availability statement

The original contributions presented in the study are included in the article/Supplementary material, further inquiries can be directed to the corresponding author.

## Author contributions

AG analyzed data and was responsible for the first draft of this manuscript. BG and MZ created the figures. XS collected the data. WJ and DT designed the research and revised the draft manuscript. All authors approved the final manuscript.
