# Supplementary material for: Bioinformatic identification of hub genes Myd88 and Ccl3 and TWS-119 as a potential agent for the treatment of massive cerebral infarction
Source: Front Neurosci. 2023 May 10;17:1171112. doi: 10.3389/fnins.2023.1171112 (PMC10206038; doi:10.3389/fnins.2023.1171112)
Supplement: Supplementary file 1 [file Table_1.DOCX]

Supplementary Material

Bioinformatic identification of hub genes *MyD88* and *Ccl3* and TWS-119 as a potential agent for the treatment of massive cerebral infarction

Ai Guo^1^, Bin Gao^1^, Mengting Zhang^1^, Xiaoyu Shi^1^, Weina Jin^2^, Decai Tian ^1^

*** Correspondence:** Decai Tian: decaitian@hotmail.com

## Supplementary Table

**Supplementary Table1.** Characteristic of selected microarray datasets

| **GSE series** | **Platform** | **Total sample** | **Selected samples (Sham/MCAO)** | **Selected tissue** | **Contributors** | **Year** |
| --- | --- | --- | --- | --- | --- | --- |
| GSE28731 | GPL1261 | 10 | 2/3 | Ipsilateral Brain | Barreto G | 2011 |
| GSE32529 | GPL1261 | 224 | 4/4 | Ipsilateral Brain | Stenzel-Poore MP | 2011 |

MCAO: Middle cerebral artery occlusion
